# Environmental barriers perceived by the Finnish population with spinal cord injury: a cross-sectional survey

**DOI:** 10.1038/s41393-024-00990-x

**Published:** 2024-04-23

**Authors:** Sanna-Mari Saarimäki, Paula Reiterä, Anni Täckman, Jari Arokoski, Aki Vainionpää, Mauri Kallinen, Susanna Tallqvist, Eerika Koskinen, Harri Hämäläinen, Anna-Maija Kauppila, Heidi Anttila, Sinikka Hiekkala

**Affiliations:** 1 Faculty of Sport and Health Sciences, University of Jyväskylä, Jyväskylä, Finland; 2 Biostatistics Unit, Department of Public Health, University of Helsinki and Helsinki University Hospital, Helsinki, Finland; 3 The Finnish Association of Spinal Cord Injured Akson, Helsinki, Finland; 4 Department of Internal Medicine and Rehabilitation / Spinal Cord Injury Outpatient Clinic, Helsinki University Hospital, Helsinki, Finland; 5 Department of Rehabilitation, Seinäjoki Central Hospital, Seinäjoki, Finland; 6 Department of Rehabilitation Medicine, Hospital Nova of Central Finland, Central Finland Health Care District, Jyväskylä, Finland; 7 Center for Life Course Health Research, University of Oulu, Oulu, Finland; 8 Faculty of Medicine, University of Helsinki, Helsinki, Finland; 9 Department of Sensory, Neural, and Musculoskeletal Medicine, Tampere University Hospital, Wellbeing Services County of Pirkanmaa, Tampere, Finland; 10 Department of Medical Rehabilitation / Spinal Cord Injury Outpatient Clinic, Oulu University Hospital, Oulu, Finland; 11 Public Health and Welfare Department, Finnish Institute for Health and Welfare (THL), Knowledge Management and Co-creation Unit, Helsinki, Finland; 12 The Finnish Association of People with Physical Disabilities, Helsinki, Finland

**Keywords:** Health care, Disability, Spinal cord diseases

## Abstract

**Study design:**

Cross-sectional survey of the Finnish population with spinal cord injury (SCI).

**Objectives:**

To explore the frequencies of perceived environmental barriers (EB) that made participation harder for the Finnish population with SCI and to compare the occurrence of perceived EBs by gender, age, time since injury, and injury severity.

**Setting:**

Participants were recruited from the registers of the three SCI outpatient clinics responsible for the lifelong care of people with SCI in Finland.

**Methods:**

The self-administered Nottwil Environmental Factors Inventory Short Form (NEFI-SF) collected in the Finnish Spinal Cord Injury Study (FinSCI) (*n* = 1772) was used. Nonparametric tests and multinomial logistic regression models were utilized.

**Results:**

880 individuals responded to the NEFI-SF items (response rate 50%). Climate was perceived as a barrier by 72% and a serious one by 44% of the respondents. The rates regarding public access were 59% and 24%, private home access 46% and 18%, and long-distance transport 45% and 20%. Four out of ten respondents reported that finances, lack of assistive devices for short-distance transport, and political decisions restricted their participation. The NEFI-SF total scores were higher (meaning more perceived restrictions by EBs) for those more severely injured.

**Conclusions:**

Climate, access to public and private places, challenges with transport, finances, and political decisions were the EBs most frequently perceived to restrict participation by the Finnish population with SCI. Most EBs that were prominent causes of restrictions are modifiable. Greater accessibility to the built environment, equal services to all, and positive special treatment could reduce their effects.

## Introduction

Disability largely results from an environment that prevents persons with impairments from fully participating in society [1]. The International Classification of Functioning, Disability, and Health (ICF) describes environmental factors as the physical, social, and attitudinal environments in which people live and conduct their lives [2]. Environmental factors, together with other ICF components, affect whether someone is able to participate in society, participation meaning involvement in a life situation [2]. Viewed from an individual’s perspective, environmental factors are either environmental barriers (EB) or environmental facilitators [2]. Close to 200 countries have committed to eliminating EBs that could hinder people with disabilities from participating in society [1].

Investigating EBs is pivotal to enabling equal and barrier-free societal participation for persons with spinal cord injury (SCI) [1]. Once the EBs causing the most restrictions are identified, it is possible to work toward minimizing their impact on the lives of people with SCI. The Nottwil Environmental Factors Inventory Short Form (NEFI-SF) was developed to assess perceived EBs in populations with SCI [3]. Few detailed NEFI-SF-based reports are available to date concerning substantial populations with SCI: The most frequently perceived EB has been the climate in Switzerland, Germany, and China and financial problems in Morocco [4–8].

Thus far, EBs that people with SCI encounter have never been researched in Finland. This study was part of the Finnish Spinal Cord Injury Study (FinSCI) [9] and aimed to discover, based on the NEFI-SF, which EBs the Finnish population with SCI perceived as making their daily life and participation in society harder. This article also compared the occurrence of perceptions of EBs by gender, age, time since injury, and severity of SCI.

## Methods

### Design

The present study aimed, as part of the mixed-method FinSCI study’s survey, to explore the occurrence of perceived EBs that complicate the daily life and participation of people with SCI in Finland (ClinicalTrials.gov number: NCT04649814). The FinSCI cross-sectional survey data were collected by mailing a 28-page self-report questionnaire to 1772 persons with SCI in 2/2019. Participants responded digitally or on paper until 7/2019 [9, 10].

### Sample

Participants were recruited from the databases of the three regional SCI outpatient clinics (Helsinki, Tampere, and Oulu University Hospitals), where the lifetime care of the population with SCI in Finland has been centered since 2011 [10: Supplementary Fig. A]. Individuals at least 16 years of age who had a non-traumatic or traumatic SCI classified with AIS grade A, B, C, or D, following the protocol of the International Standards for Neurological Classification of SCI (ISNCSCI) [11], were selected for the study. The ISNCSCI classifications were completed by several physicians and physiotherapists between 2000 and 2018. The latest classification from the medical records was used. The exclusion criteria were AIS grade E, congenital SCI, progressive and new non-traumatic SCI, neurodegenerative disease, multiple sclerosis, amyotrophic lateral sclerosis, Guillan-Barré syndrome, and living in an institution. The full protocol of the FinSCI study has been presented elsewhere [9].

### Outcome measure

EBs were assessed with the NEFI-SF—a 14-item self-report instrument linked to the environmental factors on the ICF Core Sets for SCI. It is used in population-based studies to measure the perceptions of EBs restricting the participation of people with SCI [3]. The following introduction precedes the NEFI-SF’s questions: “In daily life, one is exposed to diverse external influences (so-called environmental factors), which can make everyday easier or more difficult. Which factors made your participation in society a little, or considerably more, difficult in the last 4 weeks?” [3]. Appendix A contains the English version of the NEFI-SF.

Individual NEFI-SF items cover potential EBs concerning accessibility, attitudes, climate, communication devices, finances, medical supplies, personal care assistance, political decisions, and transport. Each item is scored “no influence” (0), “made my life a little harder” (1), or “made my life a lot harder” (2). Higher NEFI-SF total scores (range 0–28) indicate someone perceiving more restrictions due to EBs. The FACIT translation process was used for the Finnish version of the NEFI-SF [12]. The NEFI-SF is, so far, the only self-report tool designed specifically for populations with SCI whose reliability and validity have been found acceptable [3, 4, 13]. The NEFI-SF item fit has been good (mean squared errors 0.77–1.22) in a Swiss study, although floor effect (23%) and some differential item functioning related to SCI level/completeness or language were found [3]. The internal consistency of NEFI-SF items as a three-level categorical variable has been good (Cronbach’s alpha 0.82–0.87) in previous studies [3, 4]. Cronbach’s alpha was 0.85 in this Finnish sample.

### Statistical analyses

Statistical analyses were performed with IBM SPSS Statistics 28.0 (Armonk, NY, USA) and R-4.3.0 (R Foundation for Statistical Computing). Descriptive statistics are presented as frequencies and percentages or medians and interquartile ranges (IQR), depending on the variable distribution. Respondents’ and non-respondents’ demographics were compared with *χ*^2^ and Mann–Whitney tests. The data were not normally distributed; therefore, nonparametric tests were utilized. Associations of gender, age, time since injury, and ISNCSCI injury severity with the NEFI-SF total score were examined using the Mann–Whitney test, the Kruskal–Wallis test, and Spearman’s correlations. Curvilinearity was checked for age and time since injury. Dunn’s test with the Benjamini–Yekutieli procedure was used as the post hoc method for the Kruskal–Wallis test. The effect size for the Mann–Whitney and Kruskal–Wallis tests is reported as *η*^2^, and for the *χ*^2^ test as Cramér’s *V*. The correlation coefficient (*ρ)* is reported as the effect size for the Spearman correlation.

Multinomial logistic regression analyses, including estimated odds ratios (OR) with 95% confidence intervals (CI), were conducted to compare the relative frequencies of responses in the NEFI-SF items by the participant characteristics listed above. Each NEFI-SF item was modeled in a single multivariable logistic regression, including all the predictors listed in the table. A recursive partitioning analysis (CHAID method with classification tables) was performed to see whether any subgroups should be analyzed separately. The predictive power of each multinomial regression model was tested using likelihood ratio tests against an intercept-only model. The multinomial logistic regression models differentiated those respondents whose lives an EB had not influenced from those whose lives the EB had made “a little” or “a lot” harder. The AIS D group served as the reference category in injury severity comparisons. *P* values <0.05 were considered statistically significant.

**Fig. 1:**
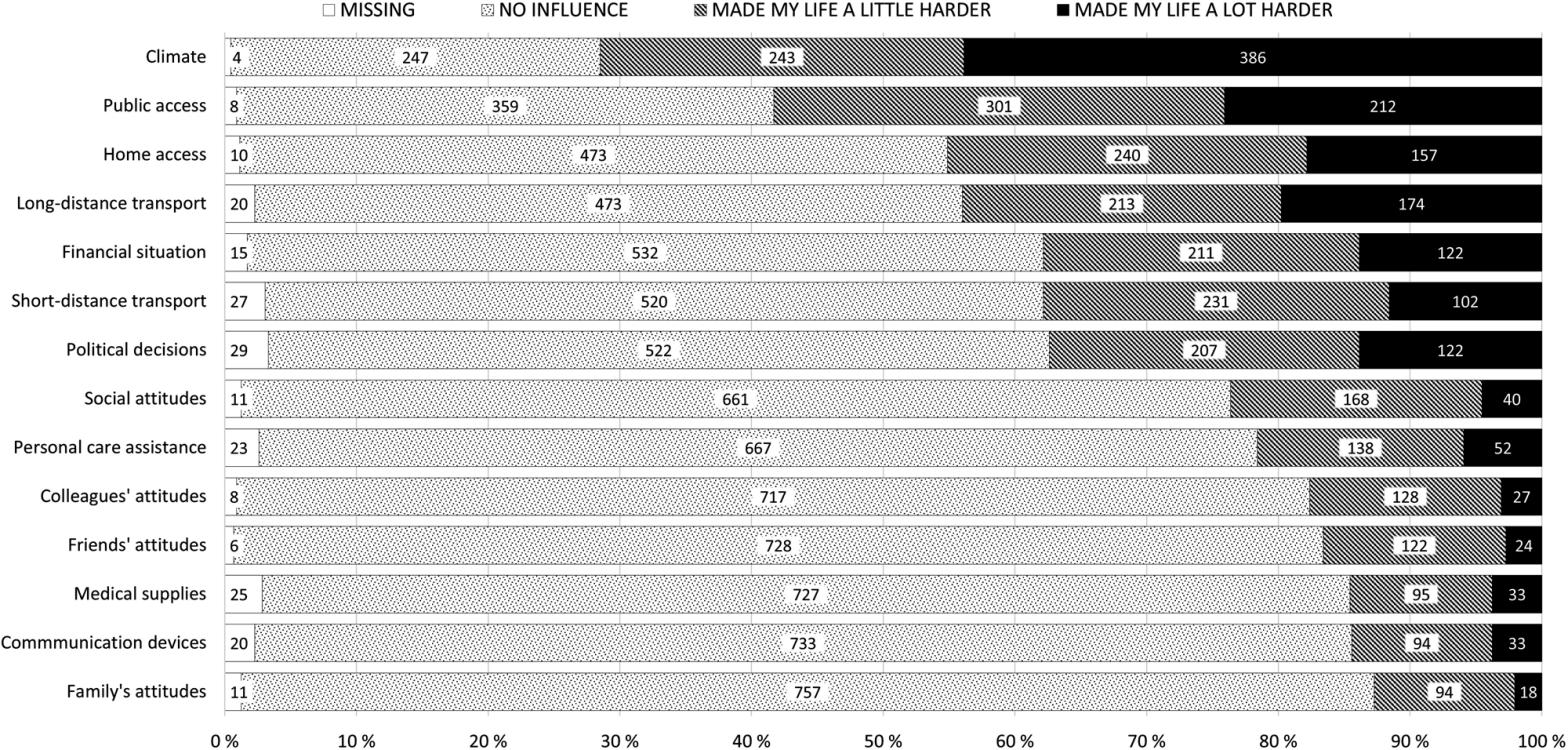
Self-reported effect of environmental barriers on the daily life of the Finnish people with spinal cord injury (FinSCI survey), *n* = 880.

Four FinSCI survey respondents who had omitted the NEFI-SF items completely were excluded from all analyses. Missing responses in the remaining NEFI-SF data (*n* = 880) varied from 4 to 29 (0.5–3.3%) per item, and no imputation was performed. Figure 1 presents the item-wise number of respondents. Seventy-one respondents (8.1%) with one or more missing items were excluded from the NEFI-SF total score analyses.

## Results

### Characteristics of respondents and non-respondents

The response rate in the FinSCI survey was 50% (*n* = 884). The NEFI-SF respondents (*n* = 880) were 66% male, and 56% had a traumatic SCI. The age of NEFI-SF respondents varied from 20 to 90; 44% were 61–75; only 4% were 30 or younger. Most respondents (62%) had an AIS D status. A majority (66%) had been injured within 10 years (counting back from their response date). Table 1 presents detailed characteristics and differences between the NEFI-SF respondents (*n* = 880) and non-respondents (*n* = 892). Comparisons between the FinSCI respondents (*n* = 884) and non-respondents (*n* = 888) have been described in earlier publications [10, 14, 15]. No statistically significant differences existed between the NEFI-SF respondents and non-respondents in SCI etiology, time since injury, or SCI severity. Statistically significant differences (*p* < 0.01) were found in gender (Cramér’s *V* = 0.068) and age (*η*^2^ = 0.04): women responded more often than men, those aged 61–75 were the most active responders, and those younger than 46 were the least active responders.

**Table 1:** Eligible population of the Finnish Spinal Cord Injury Study (FinSCI) survey (*n* = 1772) divided into NEFI-SF respondents (*n* = 880) and non-respondents (*n* = 892).

	Respondents, *n* = 880	Non-respondents, *n* = 892	*p* value	Effect size
	*n* (%)	*n* (%)		
Gender			0.004^a^	0.068 (Cramér’s *V*)
Male	574 (65)	638 (72)		
Female	306 (35)	254 (28)		
Age group	min 20, max 90, mean 61, SD 14, median 63, IQR 53–71	min 17, max 93, mean 54, SD 17, median 55, IQR 40–68	<0.001^b^	0.040 (*η*^2^)
20–30 years	34 (4)	96 (11)		
31–45 years	108 (12)	204 (23)		
46–60 years	237 (27)	244 (27)		
61–75 years	384 (44)	245 (27)		
≥76 years	117 (13)	103 (12)		
Severity of SCI			0.26^a^	0.047 (Cramér’s *V*)
C1–4 AIS A, B, C	94 (11)	108 (12)		
C5–8 AIS A, B, C	55 (6)	62 (7)		
T1–S5 AIS A, B, C	184 (21)	209 (23)		
AIS D at any injury level	547 (62)	513 (58)		
Time since injury	min 1, max 67, mean 11, SD 11, median 7, IQR 4–14	min 1, max 66, mean 10, SD 10, median 6, IQR 4–14	0.07^b^	0.002 (*η*^2^)
1–5 years	352 (40)	380 (43)		
6–10 years	225 (26)	224 (25)		
11–15 years	127 (14)	112 (13)		
≥16 years	176 (20)	176 (20)		
Etiology			0.12^a^	0.037 (Cramér’s *V*)
Traumatic	490 (56)	529 (59)		
Non-traumatic	390 (44)	363 (41)		
Life situation				
Work (full-/part-time)	115 (13)			
Sick leave/disability pension/rehabilitation subsidy/unemployed/laid off	352 (40)			
Vocational rehabilitation/student/family leave/other	40 (5)			
Old-age pension/part-time pension	372 (42)			
Missing information	1 (<1)			
Form of residence				
With a partner, no children	399 (45)			
Alone, no children	337 (38)			
With a partner and a child/children	96 (11)			
Other form of residence	45 (5)			
Missing information	3 (<1)			

^a^*χ*^2^ test.

^b^Mann–Whitney *U* test, continuous variable was used for calculations.

### Perception of environmental barriers

Figure 1 presents the frequencies of responses in individual NEFI-SF items. Table 2 shows percentages with 95% CIs. Minor (“made my life a little harder”) EBs were generally reported more often than serious (“made my life a lot harder”) ones. Most individuals had perceived difficulties due to the climate and public access. Other barriers frequently perceived as serious were long- and short-distance transport, access to the homes of friends and relatives, political decisions, and finances. Medical supplies, communication devices, and attitudes of family, friends, and colleagues were reported as barriers the least frequently. Public access was the number one barrier in the “made my life a little harder” category.

**Table 2:** Frequencies, relative percentages, and 95% CIs of the individual NEFI-SF items in the Finnish Spinal Cord Injury Study (FinSCI) survey, *n* = 880.

		No influence			Made my life a little harder			Made my life a lot harder		
	*Total n*	*n*	%	95% CI	*n*	%	95% CI	*n*	%	95% CI
Climate	876	247	28.2	25.3–31.3	243	27.7	24.8–30.9	386	44.1	40.8–47.4
Public access	872	359	41.2	37.9–44.5	301	34.5	31.4–37.8	212	24.3	21.5–27.3
Home access	870	473	54.4	51.0–57.7	240	27.6	24.7–30.7	157	18.0	15.6–20.8
Long-distance transport	860	473	55.0	51.6–58.4	213	24.8	21.9–27.8	174	20.2	17.6–23.1
Financial situation	865	532	61.5	58.2–64.7	211	24.4	21.6–27.4	122	14.1	11.9–16.6
Short-distance transport	853	520	61.0	57.6–64.2	231	27.1	24.2–30.2	102	12.0	9.9–14.4
Political decisions	851	522	61.3	58.0–64.6	207	24.3	21.5–27.4	122	14.3	12.1–16.9
Social attitudes	869	661	76.1	73.1–78.8	168	19.3	16.8–22.2	40	4.6	3.3–6.3
Personal care assistance	857	667	77.8	74.9–80.5	138	16.1	13.7–18.8	52	6.1	4.6–7.9
Colleagues’ attitudes	853	717	84.1	81.4–86.4	128	15.0	12.7–17.6	27	3.2	2.1–4.6
Friends’ attitudes	874	728	83.3	80.6–85.7	122	14.0	11.8–16.5	24	2.7	1.8–4.1
Medical supplies	855	727	85.0	82.4–87.3	95	11.1	9.1–13.5	33	3.9	2.7–5.4
Communication devices	860	733	85.2	82.6–87.5	94	10.9	9.0–13.3	33	3.8	2.7–5.4
Family’s attitudes	869	757	87.1	84.7–89.2	94	10.8	8.9–13.1	18	2.1	1.3–3.3

### NEFI-SF total score comparisons by gender, age, time since injury, and SCI severity

The NEFI-SF total score results varied from 0 to 26 (median 5, IQR 2–9). No barriers were reported by 102 individuals (Appendix B). According to the Mann–Whitney test, women had higher total scores than men (mean rank 430/392, *Z* = −2.196, *p* = 0.028). Age and NEFI-SF total score did not correlate (*ρ* = 0.04, 95% CI −0.03–0.01, *p* = 0.23). Time since injury had a very weak correlation with the NEFI-SF total score (*ρ* = 0.18, 95% CI 0.11–0.24, *p* < 0.0001). In addition, curvilinear relationships between the NEFI-SF total score and age or time since injury were not detected (Appendix C). The Kruskal–Wallis test showed differences in the NEFI-SF total score between SCI severity groups, *H* = 63.373, *p* < 0.0001; the C1–C4 AIS A, B, C group had the highest scores. The AIS D group’s total scores were lower than those of all other groups (adjusted significances *p* = 0.0025, *p* < 0.0001, *p* < 0.0001), but there were no statistically significant differences in respective pairwise comparisons between the other groups. Table 3 shows detailed results with effect sizes.

**Table 3:** **a** NEFI-SF total scores of SCI severity groups and genders in the Finnish Spinal Cord Injury Study (FinSCI) survey, *n* = 880 (NEFI-SF total score ranges 0–28, higher scores indicate more perceived restrictions due to environmental barriers); **b** The Spearman correlations between NEFI-SF total scores and age and time since injury.

a									
	*n*	Effect size (*η*^*2*^)	*p* value	Mean	95% CI	SD	Mean rank	Median	IQR
ISNCSCI group									
AIS D	498	0.075	<0.001	5.2	4.7─5.6	5.0	353.6	3	1─8
T1–S5 AIS A, B, C	173	0.075	<0.001	7.7	6.9─8.4	5.2	486.8	7	4─11
C5–C8 AIS A, B, C	50	0.075	<0.001	7.1	5.8─8.4	4.5	473.9	6	4─9
C1–C4 AIS A, B, C	88	0.075	<0.001	7.8	6.8─8.9	5.0	496.0	8	4─10
Gender									
Male	528	0.006	0.028	5.8	5.4─6.2	5.0	391.9	5	2─9
Female	281	0.006	0.028	6.7	6.1─7.4	5.5	429.7	6	2─11
Total	809			6.1	5.8─6.5	5.2		5	2─9

b			
	*n*	Effect size (*ρ*)	*p* value
Age	809	0.04	0.23
Time since injury	809	0.18	<0.001

### NEFI-SF item-wise comparisons by gender, age, time since injury, and SCI severity

No evident subgroup construct was found in the recursive partitioning analysis, and the data were used as a whole in the multinomial logistic regression analyses. Table 4 indicates the ORs and frequencies of perception of each NEFI-SF item by gender, age, time since injury, and SCI severity. For a more comprehensive picture, the results are provided with two reference groups: “no influence” and “a little harder”.

**Table 4:** **a** Multinomial logistic regression models of individual NEFI-SF items in the Finnish Spinal Cord Injury Study (FinSCI) survey, *n* = 880; “NO INFLUENCE” as a reference group; **b** Multinomial logistic regression models of individual NEFI-SF items in the Finnish Spinal Cord Injury Study (FinSCI) survey, *n* = 880; “MADE MY LIFE A LITTLE HARDER” as a reference group.

**a**	
**b**	

Please note that the variables have different units of measurement, so the ORs cannot be directly compared between the variables.

The item “family’s attitudes” is not presented as it was statistically not a good fit (Pearson’s *χ*^2^ statistic *p* = 0.004).

Statistically significant *p* values are highlighted with darker shades (the greater the significance, the darker the shade): *p* < 0.05, *p* < 0.01, *p* < 0.001.

An odds ratio is the division of an odds by another odds. The OR is interpreted as the change in the odds for each increase of one unit of the independent variable. For example, the model explaining perceived EBs regarding public access—the comparison of “a lot harder” to “a little harder”: For age, an increase of one unit (being one year older) corresponds to 1.04-fold odds compared to someone one year younger, meaning the odds of reporting “a lot harder” are 4% larger for someone one year older as opposed to if they were a year younger. Values <1 indicate decreased odds for an increase of one unit of the independent variable. Please note that the variables have different units of measurement, so the ORs cannot be directly compared between the variables.

There were only a few responses for some items in the “a lot harder” group but many in the “no influence” group, affecting model fit. Nevertheless, with a statistically significant Pearson’s *χ*^2^ statistic (*p* < 0.01), the family’s attitudes was the only item in which the model was statistically not a good fit. According to the likelihood ratio tests, despite some statistically significant parameter estimates, the multinomial logistic regression model did not explain responses in the colleagues’ attitudes (*p* = 0.20) and medical supplies (*p* = 0.25) items.

## Discussion

This study investigated the perceived EBs in the Finnish population with SCI for the first time and analyzed the responses by gender, age, time since injury, and SCI severity. The climate was an outstanding barrier, the reason 72% of the respondents reported difficulties in daily life and the leading EB perceived to cause serious restrictions. Public access was another serious barrier and most often perceived to make life “a little harder”. Problems with accessing private homes and long-distance transport reportedly restricted the participation of nearly half the respondents. Short-distance transport, finances, and political decisions were considered barriers by four out of ten respondents. Individuals with more severe injuries (groups C1–C4 AIS A, B, C; C5–C8 AIS A, B, C; and T1–S5 AIS A, B, C) perceived more restrictions because of EBs than respondents with AIS D classification; these differences were highlighted in the items reported as barriers most frequently. Most EBs that stood out as prominent causes of restrictions are modifiable. Attention should be paid to reducing their effects on the lives of people with SCI.

It was positive that the “no influence” group was the largest for many items, and the “a lot harder” answers constituted a small minority. Extensive comparisons with other studies using the NEFI-SF are challenging due to dissimilar methods; for example, many studies have used the total number of barriers (barrier vs. no barrier) in their analyses [5, 7, 16–18]. However, the Finnish individuals with SCI seemingly perceived fewer EBs than those with SCI in some other countries [4, 5, 16, 17]. For instance, on a dichotomized scale, the Finns reported fewer EBs than the respondents of the International Spinal Cord Injury Survey (InSCI) sample of 22 countries (FinSCI: median 4, IQR width 5; InSCI: median 5, IQR width 6). On average, the most relevant EBs restricting life were the same as in the InSCI, as were the least reported barriers.

Our results resemble those found in Switzerland and Germany; there were minimal differences in the order of importance of the barriers reported the most in all three countries [3, 6–8]. Many EBs have generally been observed in countries with low resources [4, 16]; accordingly, it is no surprise that the distributions of perceived EBs are similar in the high-income European countries. Nonetheless, the Finnish people with SCI answered “a lot harder” statistically significantly more frequently than their European colleagues for most of the same top seven items in those countries (Appendix D) [3, 8].

The climate—the most frequently perceived EB among the FinSCI respondents—has been viewed as a remarkable barrier in many populations [5–8, 18–22]. Weather extremes are common in Finland, which likely explains the strikingly high percentage of participants regarding the climate as a serious barrier. Over three-quarters of the FinSCI participants submitted their responses by the end of March; hence, the survey’s timing during snowy months may have highlighted the relevance of climate in their opinions. Snow and ice make surfaces uneven and slippery, often restricting or blocking walking and moving in a wheelchair [19, 21, 22]. Furthermore, SCI affects thermosensitivity and -body regulation, especially in those more severely injured [23]. Weather and temperatures cannot be controlled. Nevertheless, the blocking impact of snow and ice can be actively lessened during Finland’s characteristically long winters. This could be done, for instance, by taking care of snowplowing and clearing public places (e.g., roads, pedestrian areas, building entrances, parking lots, public transport stops, and curb cuts) of barriers [21, 22].

Long-distance transport was perceived to pose challenges for almost half the respondents and serious difficulties for a fifth. Living far from community centers often means long rides to access health care and amenities and has been linked to perceived activity limitations in persons with SCI, which may result in functional decline [24]. In Finland, 5.5 million people live in an area circa the size of Germany. Consequently, traveling extended distances is necessary for many, especially after centralizing medical care for people with SCI. Vehicle ownership has been associated with satisfaction with transport [25]. Lack of adapted vehicles (or other forms of reliable, accessible, and frequently available transport), shortage of suitable parking, and the high cost of motoring in Finland might be some reasons Finnish people with SCI considered moving long distances an outstanding barrier [20–22, 24–27].

Challenges with public and private home accessibility and the lack of adapted assistive technology for moving short distances were perceived to cause significant participation difficulties for the respondents. Finances and political decisions were other issues viewed as hampering the lives of a noticeable part of our sample—common discoveries in studies on EBs and participation by people with SCI [4–8, 16, 17, 19–21, 24–28]. Society can influence all these matters, and steps could be taken to address them. The needs of people with disabilities should be considered through legislative and other measures, such as civil engineering, to develop policies, infrastructure, and transport that is friendlier and more equal to all citizens. Social attitudes and support are some of the strongest environmental factors enhancing or restricting participation [19–21, 25–28]. Other people’s attitudes were among the least reported barriers in this study. The stances toward people with SCI in Finland may be relatively neutral or even encouraging. This might play a part in the respondents’ low total score of perceived barriers.

The results of the statistical analyses were unsurprising. Severely injured people reported EBs as restricting their lives more than those with milder injuries, which was highlighted in the items most frequently perceived to make life harder. Similar observations have been made in some earlier research in which having complete SCI has been associated with perceiving more barriers, but the evidence is not solid [6, 8, 16]. In our sample, the climate was perceived to produce statistically significantly more serious but not minor difficulties in the two groups of the highest injury level (compared with the AIS D group). It is conceivable that the climate was a serious barrier for them for numerous reasons; therefore, few consider it a minor restriction. Women reported restrictions by EBs more often than men. The effect size remained small, calling into question the result’s clinical relevance; however, the result aligns with previous research [16]. Those who had been injured longer had slightly higher NEFI-SF total scores, although the clinical relevance is negligible with the small correlation coefficient. Even so, any of these identified effects are at the population level and may not capture differing patterns within subgroups. Understanding such subpopulation patterns is a valuable avenue for future research.

### Strengths and limitations

Our study’s sample was contacted utilizing the patient databases of all three specialized SCI clinics in Finland—the only three centers decreed by the Finnish government since 2011 to offer services for the whole SCI population, including people injured before 2011 [10: Supplementary Fig. A]. This enabled us to reach most of the target group and compare the generic and lesion characteristics between respondents and non-respondents. Nevertheless, long-term injuries may be underrepresented in the registers. Another advantage of this study was that the SCI severity of all participants had been classified according to the ISNCSCI, as the International Spinal Cord Injury Core Data Set (version 2.0) recommends [29].

The 50% response rate contains the possibility of response bias, and we can see from the registered data that young people did not respond often. Additionally, people needing help completing questionnaires may be missing; however, injury severity comparisons do not indicate this. Nevertheless, the response rate was adequate for this type of extensive questionnaire, and the number of respondents was sizeable compared to similar studies performed in more populous countries [30]. The sparsity of missing responses in the NEFI-SF items further increases the reliability of our results. This article concentrated solely on analyzing the NEFI-SF responses, allowing for specific analyses of individual NEFI-SF items. Yet, a limitation is that this study focuses exclusively on EBs without analyzing interactions between different parts of the FinSCI questionnaire (e.g., activity and participation).

Statistical analyses were performed using the original three-class scale (“no influence”/“a little harder”/“a lot harder”), contrary to some studies, in which the NEFI-SF answers were dichotomized or combined for analyses, or only descriptive results were presented. However, skewness of responses undermines the reliability of the logistic regression models in three items, and the pseudo-*R*^2^ statistics are low, suggesting the models explained only some of the variation in reported EBs. This implies that factors other than those this study focused on are important for predicting perceived EBs. Because each question was analyzed separately, a type I error (some variable appearing statistically significant by chance) is possible.

The results of the multinomial regressions should be interpreted with caution. The models lack power when, in many items, only a small percentage of respondents perceived them as barriers. The large group choosing “no influence” may consist of people having varying reasons for being unaffected (e.g., they may have good functioning, stay home because of poor functioning, or be positive people less inclined to complain about things). Lacking positive answer categories, the NEFI-SF does not measure the possible beneficial effects of environmental factors on participation. Also, the four-week timeframe of the measure is short, which may have a bearing on the high frequency of “no influence” responses. More psychometric studies on the NEFI-SF are needed.

The female gender and older age categories are slightly over-represented in our sample, although the effect sizes were very small. The possibility of our models not reaching high predictive power because of the biased sample cannot be excluded; it is uncertain that nonsignificant results are due to a real absence of effect. More advanced statistical analyses could be done to explore the matter thoroughly, considering the respondents’ different background characteristics, such as family situation.

The reliability of self-assessment is debatable, especially considering the paradox of barriers [31]: Individuals who potentially would encounter the most barriers restricting participation may avoid activities and situations in which they would be exposed to barriers; thus, reduced participation results in fewer reported barriers. Conversely, those in a better position to overcome barriers generally attempt to participate more often; hence, they become more exposed to the EBs through participation, which generates reporting more barriers. Over 10% of the respondents reported no problems, which could partly be the paradox of barriers at work. Other explanations might be the floor effect of the NEFI-SF [3] or those people reporting no barriers may have good functionality, just as the AIS D group (containing the most respondents) reported fewer restrictions due to EBs than other respondents.

## Conclusions

Our study provides the first information about EBs complicating the lives of people with SCI in Finland. The climate, public and private access, and long-distance transport were the most frequently perceived barriers to participation in the Finnish population with SCI, followed by finances, short-distance transport, and political decisions. People with more severe injuries reported more restrictions due to EBs. Society should strive to reduce the impact of EBs on the lives of Finnish people with SCI. Decisionmakers can utilize this data when planning which EBs require the most actions to enable equal, barrier-free participation in society for people with SCI in Finland.

### Supplementary information

Appendices A–D

## Data Availability

The datasets generated and analyzed during the current study are available from the corresponding author upon reasonable request.
