# Supplementary material for: Environmental barriers perceived by the Finnish population with spinal cord injury: a cross-sectional survey
Source: Spinal Cord. 2024 Apr 23;62(6):348–55. doi: 10.1038/s41393-024-00990-x (PMC11199142; doi:10.1038/s41393-024-00990-x)
Supplement: Supplementary file 1 — Appendices A–D [file 41393_2024_990_MOESM1_ESM.pdf]

TITLE: Environmental barriers perceived by the Finnish population with spinal cord injury: a cross-sectional survey

AUTHORS: Sanna-Mari Saarimäki, Paula Reiterä, Anni Täckman, Jari Arokoski, Aki Vainionpää, Mauri Kallinen, Susanna Tallqvist, Eerika Koskinen, Harri Hämäläinen, Anna-Maija Kauppila, Heidi Anttila, Sinikka Hiekkala

JOURNAL: Spinal Cord

YEAR: 2024

CONTENTS

Appendix A: English version of the Nottwil Environmental Factors Inventory Short Form (NEFI-SF) according to Ballert CS, Post MW, Brinkhof MW, Reinhardt JD. Psychometric properties of the Nottwil Environmental Factors Inventory Short Form. Arch Phys Med Rehabil. 2015; 96: 233–240. 2

Appendix B: The NEFI-SF total score frequencies of the Finnish Spinal Cord Injury Study (FinSCI) survey presented on the converted scale of 0–100 according to the conversion table by Ballert et al. 2015. 4

Appendix C: Curvilinearity of age and time since injury related to the NEFI-SF total score in the Finnish Spinal Cord Injury Study (FinSCI) survey, *n* = 809. 5

Appendix D: Percentages of the NEFI-SF answers in the Finnish (FinSCI: Saarimäki et al. 2024, *n* = 880)**, Swiss** (SwiSCI: Ballert et al. 2015, *n* = 1549), **and German** (GerSCI: Bökel et al. 2020, *n* = 1479) **samples.** 6

Appendix A: English version of the Nottwil Environmental Factors Inventory Short Form (NEFI-SF) according to Ballert CS, Post MW, Brinkhof MW, Reinhardt JD. Psychometric properties of the Nottwil Environmental Factors Inventory Short Form. Arch Phys Med Rehabil. 2015; 96: 233–240.

INTRODUCTION

In daily life, one is exposed to diverse external influences (so-called environmental factors), which can make everyday easier or more difficult.
Which factors made your participation in society a little, or considerably more, difficult in the last 4 weeks? Please consider how you would like your participation to be.

QUESTIONS

Public access: Inaccessible or inadequately accessible public places (eg, public buildings, parks)

No influence	(0)
Made my life a little harder	(1)
Made my life a lot harder	(2)

Climate: Unfavorable climatic conditions (eg, weather, season, temperature, humidity)

No influence	(0)
Made my life a little harder	(1)
Made my life a lot harder	(2)

Social attitudes: Negative societal attitudes toward persons with disability (eg, prejudice, ignorance)

No influence	(0)
Made my life a little harder	(1)
Made my life a lot harder	(2)

Attitudes of family: Negative attitudes of your family and relatives with regard to your disability (eg, prejudice, lack of support, overprotective behavior)

No influence	(0)
Made my life a little harder	(1)
Made my life a lot harder	(2)

Attitudes of friends: Negative attitudes of your friends with regard to your disability (eg, prejudice, lack of support, overprotective behavior)

No influence	(0)
Made my life a little harder	(1)
Made my life a lot harder	(2)

Attitudes of colleagues: Negative attitudes of neighbors, acquaintances, and work colleagues with regard to your disability (eg, prejudice, lack of support, overprotective behavior)

No influence	(0)
Made my life a little harder	(1)
Made my life a lot harder	(2)

Short-distance transportation: Lack of, or inadequate, adapted assistive technology for moving around over short distances. (eg, stair lift, walking aids)

No influence	(0)
Made my life a little harder	(1)
Made my life a lot harder	(2)

Long-distance transportation: Lack of, or inadequate, adapted means of transportation for long distances (eg, no adapted car, hard to use public transport)

No influence	(0)
Made my life a little harder	(1)
Made my life a lot harder	(2)

Personal care assistance: Lack of, or inadequate, nursing care and support services (eg, home health care, personal assistance)

No influence	(0)
Made my life a little harder	(1)
Made my life a lot harder	(2)

Medical supplies: Lack of, or insufficient, medication and medical aids and supplies (eg, catheters, disinfectants, splints, pillows)

No influence	(0)
Made my life a little harder	(1)
Made my life a lot harder	(2)

Financial situation: Problematic financial situation (eg, shortage of money, lack of governmental support)

No influence	(0)
Made my life a little harder	(1)
Made my life a lot harder	(2)

Communication devices: Lack of, or inadequate, communication devices (eg, writing devices, computer, telephone, mouse)

No influence	(0)
Made my life a little harder	(1)
Made my life a lot harder	(2)

Home access: Inaccessibility, or inadequate, accessibility to the homes of friends and relatives

No influence	(0)
Made my life a little harder	(1)
Made my life a lot harder	(2)

Political decisions: Inadequate national and cantonal political decisions and governmental services (eg, problems with disability insurance, lack of equality promotion)

No influence	(0)
Made my life a little harder	(1)
Made my life a lot harder	(2)

Appendix B: The NEFI-SF total score frequencies of the Finnish Spinal Cord Injury Study (FinSCI) survey presented on the converted scale of 0–100 according to the conversion table by Ballert et al. 2015.

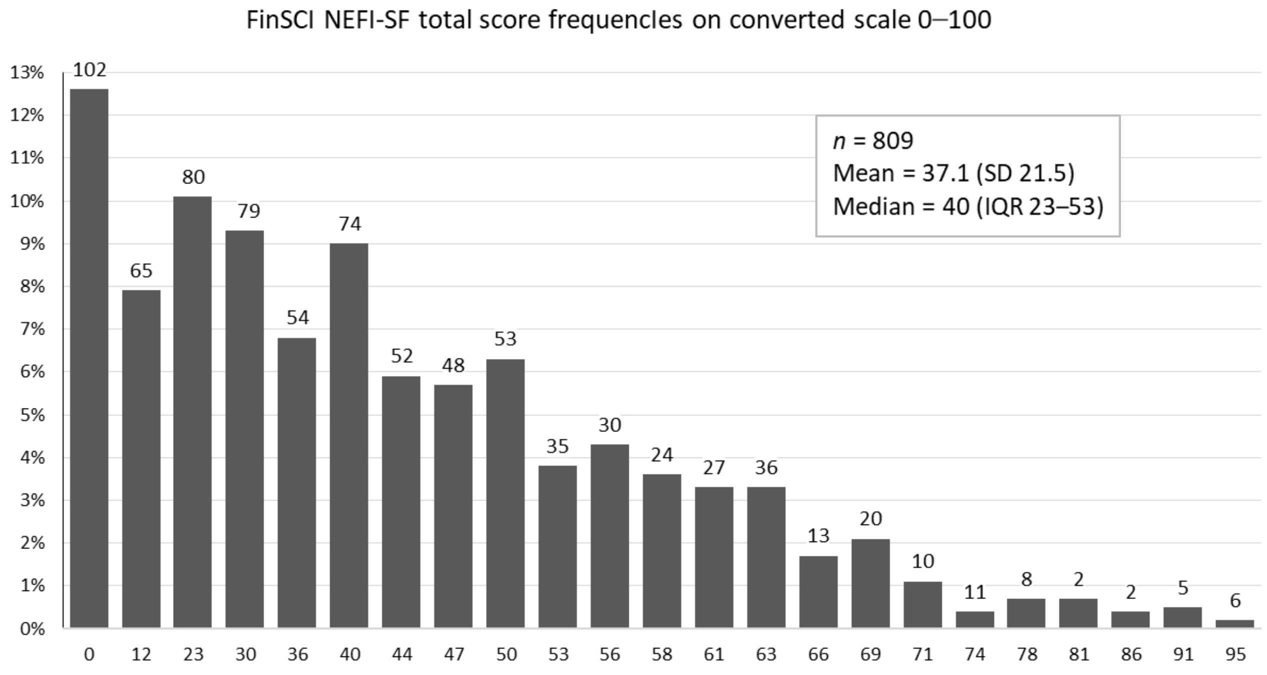

Reference

Ballert CS, Post MW, Brinkhof MW, Reinhardt JD. Psychometric properties of the Nottwil Environmental Factors Inventory Short Form. Arch Phys Med Rehabil. 2015; 96: 233–240.

Appendix C: Curvilinearity of age and time since injury related to the NEFI-SF total score in the Finnish Spinal Cord Injury Study (FinSCI) survey, *n* = 809.

For the age, there was no curvilinearity (age *β* -,266, *p* = 0.257; age^2 *β* = 0.308, *p* = 0.190), but for the time since injury, statistically significant curvilinearity was found (time since injury *β* = 0.319, *p* = 0.003; time since injury^2 *β* = -0.235, *p* = 0.026). However, sporadic observations seem to cause this curvilinearity (please see the picture below).

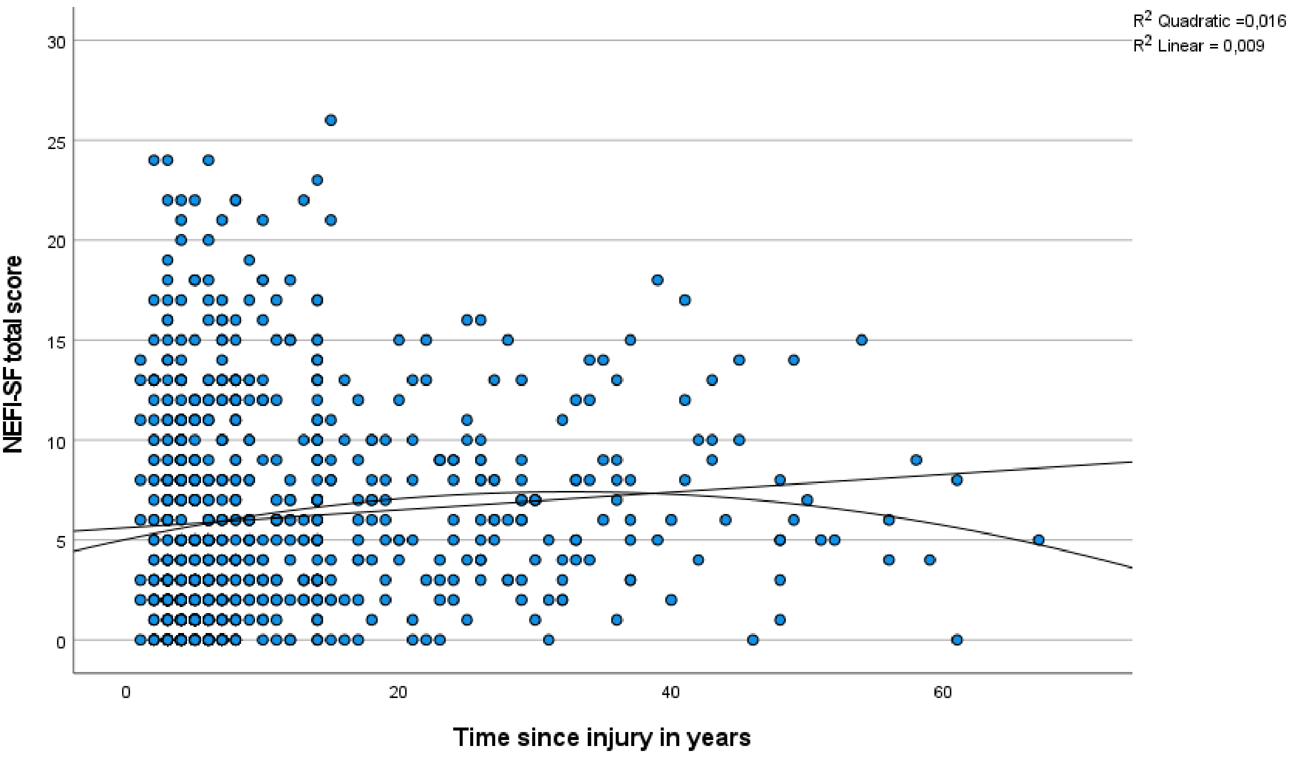

Based on both statistical and visual examination, we feel curvilinearity remains minor.

Appendix D: Percentages of the NEFI-SF answers in the Finnish (FinSCI: Saarimäki et al. 2024, *n* = 880)**, Swiss** (SwiSCI: Ballert et al. 2015, *n* = 1549), **and German** (GerSCI: Bökel et al. 2020, *n* = 1479) **samples.**

	**No influence**			**Made my life a little harder**			**Made my life a lot harder**		
	FinSCI %	SwiSCI %	GerSCI %	FinSCI %	SwiSCI %	GerSCI %	FinSCI %	SwiSCI %	GerSCI %
**Climate**	28.2	29.8	34.8	27.7	48.5	43.8	44.1	21.6	21.4
**Public access**	41.2	39.8	41.1	34.5	44.8	39.4	24.3	15.4	19.5
**Home access**	54.4	53.0	36.1	27.6	35.8	36.2	18.0	11.2	27.7
**Long-distance transport**	55.0	63.6	59.2	24.8	26.3	24.0	20.2	10.1	16.8
**Financial situation**	61.5	70.3	72.7	24.4	21.0	18.7	14.1	8.7	8.6
**Short-distance transport**	61.0	63.3	68.8	27.1	28.4	21.8	12.0	8.3	9.4
**Political decisions**	61.3	63.1	70.1	24.3	26.3	19.4	14.3	10.7	10.5
**Social attitudes**	76.1	76.6	78.0	19.3	20.0	17.4	4.6	3.4	4.6
**Personal care assistance**	77.8	87.4	86.5	16.1	9.9	9.5	6.1	2.7	4.0
**Colleagues' attitudes**	84.1	79.4*	87.5	15.0	18.5*	10.6	3.2	2.1*	2.0
**Friends' attitudes**	83.3	79.4*	89.2	14.0	18.5*	8.7	2.7	2.1*	2.0
**Medical supplies**	85.0	85.5	86.7	11.1	12.1	10.4	3.9	2.5	2.8
**Communication devices**	85.2	90.6	91.6	10.9	7.3	6.4	3.8	2.1	2.0
**Family's attitudes**	87.1	79.4*	89.2	10.8	18.5*	8.5	2.1	2.1*	2.3

* Values shown of a variable "attitudes of close persons" in which the original items "attitudes of colleagues", "attitudes of friends", and "attitudes of family" were joined.

In the top seven environmental barriers, the Finnish people with SCI chose the "a lot harder" option statistically significantly more often ($p < 0.01$) than the people in Switzerland and Germany, except for the Germans in the items of "home access" and "short-distance transport".

The statistical significance was analyzed by proportion test: prop.test-function in the R software.

References

Ballert CS, Post MW, Brinkhof MW, Reinhardt JD. Psychometric properties of the Nottwil Environmental Factors Inventory Short Form. Arch Phys Med Rehabil. 2015; 96: 233–240.

Bökel A, Dierks ML, Gutenbrunner C, Weidner N, Geng V, Kalke YB et al. Perceived environmental barriers for people with spinal cord injury in Germany and their influence on quality of life. J Rehabil Med. 2020; https://doi.org/10.2340/16501977-2717.

Saarimäki SM, Reiterä P, Täckman A, Arokoski J, Vainionpää A, Kallinen M et al. Environmental barriers perceived by the Finnish population with spinal cord injury: a cross-sectional survey. Spinal Cord 2024.
